# Nutrition-Education-Based Interventions in Gestational Diabetes: A Scoping Review of Clinical Trials

**DOI:** 10.3390/ijerph191912926

**Published:** 2022-10-09

**Authors:** Macy M. Helm, Kenneth Izuora, Arpita Basu

**Affiliations:** 1 Department of Kinesiology and Nutrition Sciences, School of Integrated Health Sciences, University of Nevada, Las Vegas, NV 89154, USA; 2 Section of Endocrinology, Department of Internal Medicine, University of Nevada, Las Vegas, NV 89154, USA

**Keywords:** gestational diabetes, self-management education, medical nutrition therapy, nutrition, health education

## Abstract

Cases of diabetes mellitus have seen a global increase in prevalence, but there are inherent differences in the pathology and management of different types of diabetes. Type 2 and gestational diabetes have the most similar pathophysiology. For that reason, many similar management strategies exist between type 2 and gestational diabetes, including nutrition-based interventions. Diabetes self-management education and medical nutrition therapy have been advanced as cost-effective interventions to manage hyperglycemia. Many of these interventions, however, were designed for type 2 diabetes and adapted for diabetes in gestation. Nutrition-education-based interventions in gestational diabetes have not been fully elucidated. We scrutinized this gap by conducting a scoping review of recently published peer-reviewed studies that evaluated clinical endpoints in cases of gestational diabetes with nutrition-education-based interventions. The search yielded 621 articles, and the 12 articles included were published between 2012 and 2022. The nutrition information varied across the heterogeneous diabetes self-management education, whereas the medical nutrition therapy studies were more consistent. Our literature search revealed similar outcomes across self-management education and medical nutrition therapy interventions implemented during the third trimester of pregnancies with diabetes. These results suggest that both generalized and personalized approaches to nutrition education in gestational diabetes can manage hyperglycemia and offset its adverse consequences.

## 1. Introduction

Globally, the prevalence and incidence of diabetes mellitus continue to increase while research strives to strengthen pharmacological and lifestyle approaches to mitigate the pathophysiology and improve disease outcomes. Estimations of diabetes prevalence often report data about the two more common classes of diabetes mellitus, type 1 (T1DM) and type 2 (T2DM). A 2019 analysis of national health surveys and databases encompassing 211 countries and territories estimated that 9.3% of the global adult population (463 million) live with T1DM and T2DM [1]. Epidemiologic data suggest gestational diabetes mellitus (GDM) occurred in 15.8% of global pregnancies in 2019 [2]. Interestingly, the changing diagnostic criteria of GDM have challenged the ability to look at trends over time to determine if GDM prevalence is increasing similarly to T2DM. Due to these challenges and a lack of global data available for analysis, projections of future GDM prevalence are uncertain. Different statistical models suggest the possibility of an increase, decrease, or stabilization of GDM [2].

Similar to all classes of diabetes mellitus, hyperglycemia due to endocrine metabolic dysfunction typifies GDM, which is the first onset of diabetes during pregnancy. The pathophysiology of GDM is similar to T2DM due to its onset being unrelated to autoimmune disease. The dysfunction of β cells, which are responsible for insulin production and release in the pancreas, is a significant component of GDM pathophysiology and is intensified by insulin resistance [3]. β-cell dysfunction is complex and varied but ultimately results in the cells’ inability to compensate for pregnancy demands [3]. This failure may contribute to the development of insulin resistance. Simultaneously, reduced insulin signaling capacity and altered insulin receptors may factor into the increased insulin resistance reported in GDM. Together, these critical metabolic disruptions result in hyperglycemia during pregnancy, subsequently increasing the risk for perinatal complications and postpartum cardiometabolic disease [3,4,5].

The current literature supports many management strategies for T2DM, which have been extended to GDM due to similar risk factors and pathophysiology. Due to the opportunity for risk modification, lifestyle behaviors are the primary focus of preventative measures. Studies have reported an average 1–2% reduction in hemoglobin A1c (HbA1c) due to nutrition therapy, and these reductions occur in newly diagnosed and long-duration cases, as well as in T1DM and T2DM [6]. Most nutrition recommendations center on a consistent distribution of carbohydrate intake [7]. Individualized nutrition recommendations, or medical nutrition therapy (MNT) conducted by registered dietitian nutritionists, are promoted as one of the more effective management techniques [6,8]. Considering the significant role diet plays in glucose metabolism, MNT is deemed foundational to the management of diabetes, especially GDM [9]. MNT utilizes the nutrition care process to complete a thorough nutrition assessment, diagnose nutrition-related problems, develop appropriate interventions and goals with the patient, and monitor and evaluate the impacts of these interventions [10]. To supplement MNT, emerging nutrition research has begun testing the role of other nutrients in the management and potential prevention of GDM. These interventions include dietary supplements, probiotics, and antioxidants; however, reported data have not yet conclusively resulted in dietary recommendations for one or more of these compounds in managing GDM [11].

To complement the recommendation of individualized nutrition therapy and support individuals with diabetes mellitus who may not have access to MNT, diabetes self-management education (DSME) programs exist as means to facilitate diabetes-related skill development and self-efficacy [12]. DSME programs are more frequently designed for T1DM and T2DM and have been reported to be cost-effective interventions that improve health outcomes globally [12]. Despite the differences between GDM and T1/T2DM, the role of DSME programs in improving self-management is hypothesized to be similar. In addition, considering the reported long-term outcomes of DSME for T2DM (i.e., improved HbA1c, quality of life, and physical activity participation) [12], these programs may also modulate the risk for postpartum cardiometabolic disease and long-term diabetes risks in women diagnosed with GDM.

Due to the current dearth of GDM-specific research, DSME programs for GDM have not been fully elucidated. Similarly, while MNT is considered the foundation of diabetes management, the current body of literature reporting the impacts of MNT in women with GDM is scarce. The management of GDM is imperative for maternal and fetal health, and both MNT and DSME programs have been reported to effectively improve hyperglycemic management and mitigate the risk of secondary complications. The significance of lifestyle modifications in GDM management underscores the importance of nutrition education integration into DSME programs when individualized nutrition therapy is not available. Thus, we conducted the present scoping review to synthesize and describe recent clinical trials on nutrition education in GDM management, both in the form of DSME and MNT. By including both categories of nutrition-based interventions, we were able to compare clinical endpoint outcomes and identify similarities in educational frameworks to elicit behavior change and optimal GDM management.

## 2. Materials and Methods

M.M.H. executed a literature search using the databases PubMed Central, Medline, and Cochrane Library. M.M.H. hand-searched for additional articles using the search engine Google Scholar. Hand-searching allowed for additional articles to be examined based on the inclusion criteria using the title, abstract, and full text when necessary. All articles were collated for data collection and first reviewed for eligibility by M.M.H. A.B. assisted in determining the search criteria and reviewing selected articles to confirm the eligibility criteria were met.

Selected articles were clinical trials that either examined the effects of DSME programs specific to GDM with a nutrition component or MNT interventions for women with GDM. All selected articles met the National Institute of Health’s definition of clinical trials. Therefore, trials involved human subjects who were prospectively assigned to an intervention, and the researchers evaluated the effects of the interventions on health-related biomedical and/or behavioral outcomes [13]. To synthesize the most recent literature, all selected articles were published between 2012 and 2022. Search terms included “gestational diabetes mellitus”, “self-management education”, “nutrition”, “healthy eating”, “lifestyle”, “medical nutrition therapy”, and “individualized nutrition”.

Inclusion criteria included: (1) human subjects with GDM; (2) DSME intervention implemented while pregnant; (3) the inclusion of a nutrition- or healthy-eating-based component of the DSME intervention; (4) MNT intervention implemented while pregnant; (5) prospective assignment to an intervention beyond standard clinical care; (6) the measurement of clinical endpoints (e.g., blood glucose, glycated hemoglobin, and gestational weight gain). Exclusion criteria included: (1) non-human models; (2) subjects with T1DM or T2DM before the GDM diagnosis; (3) subjects with co-morbidities; (4) non-self-management education intervention; (5) the omission of nutrition or healthy eating in the DSME intervention; (6) a non-MNT intervention; (7) interventions with additional pharmacology; (8) a retrospective analysis. For this review, the DSME interventions had to follow the curriculum, support, and progress standards outlined by the American Diabetes Association [14].

**Figure 1:**
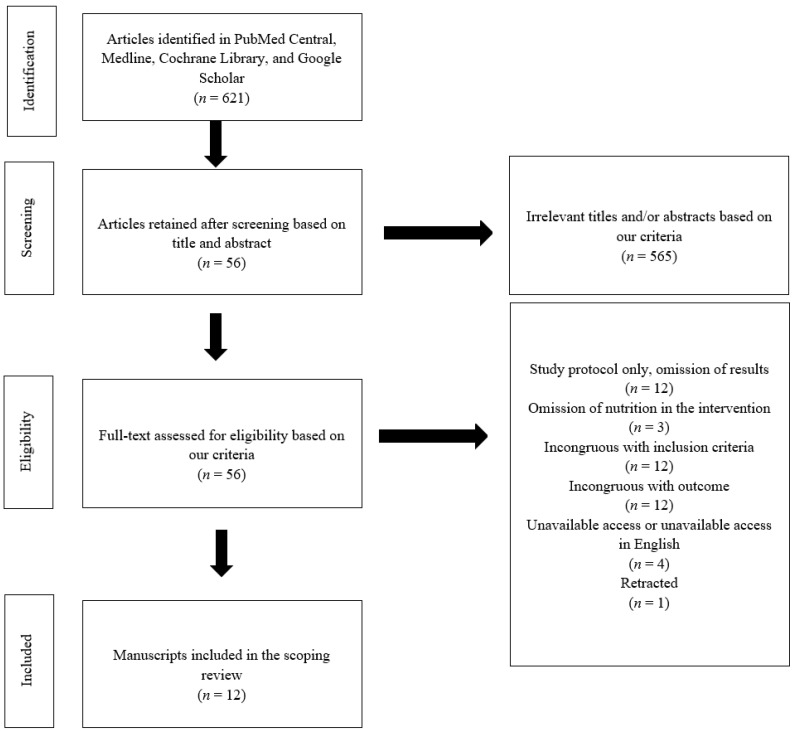
Flow diagram illustrating the search and selection of published manuscripts using the agreed-upon inclusion and exclusion criteria.

This search yielded 621 articles (Figure 1), which were assessed first by their title and abstract, and then by the subject’s diabetes status and the nutrition-based intervention, as per our predetermined eligibility criteria. Articles that aligned with these initial criteria were then fully reviewed by examining the text. In total, 12 articles were eligible and included, which was agreed upon by all authors.

The DSME interventions were evaluated by the level of detail reported on the nutrition education provided and the proportion of the overall education devoted to nutrition. The clinical endpoint outcomes of both the DSME and MNT interventions were compared to determine if one intervention classification is better suited for promoting normoglycemia in cases of GDM. Finally, the underlying frameworks of both interventions were also assessed to determine similarities and/or differences in nutrition education and the theories to motivate behavior change related to dietary intake.

## 3. Results

Of the 12 manuscripts included, all were randomized controlled trials and met the NIH definition of a clinical trial [13].

### 3.1. Diabetes Self-Management Education

As Table 1 indicates, eight studies assessed clinical endpoints related to DSME interventions [15,16,17,18,19,20,21,22,23]. The detail of the nutrition education varied between manuscripts; however, the majority of them (67%) included specifics regarding carbohydrate-based education related to carbohydrate exchange [18], sugar intake [17,19,22], fiber intake [15,16], fruit and vegetable intake [17,19], or the glycemic index of foods [15]. Despite the varied level of detail explaining the nutrition education across the manuscripts, each study met the necessary nutrition component of the DSME standards outlined by the American Diabetes Association [14]. In addition to nutrition, all DSME interventions included blood glucose self-monitoring [15,16,17,18,19,20,21,22,23].

Two studies utilized the health belief model as the framework of behavior change, and each had a component of education support outside of the structured education sessions. The first incorporated a smartphone app into usual care (a 1.5 h group education, a 1 h individual session, and subsequent support regarding diet changes based on blood glucose management) [18]. The smartphone app included health belief theory principles such as perceived threat, barriers, benefits, and self-efficacy as well as self-paced modules with more detailed lessons for DSME [18]. The primary clinical endpoint of this intervention was gestational weight gain, and the secondary clinical endpoints included glycemic control [18]. The smartphone app did not have a statistically significant impact on excessive gestational weight gain [18]. That said, the use of the app lowered the proportion of glucose above the target range before meals and two hours after meals by 26% and 38%, respectively, when compared to the control group [18]. Despite not impacting gestational weight gain, the intervention appeared to still yield positive effects on glycemic control, as indicated by the lower readings of blood glucose. Pertaining to the health belief model, the study measured the frequency of weekly blood glucose self-management, which was not statistically different between the two groups [18]. The second study incorporated phone calls to serve as a check-in and morale booster integrated into the four education sessions [20]. The intervention integrated health belief theories by discussing perceived susceptibility to GDM, the severity of GDM, the barriers and benefits of self-management, and self-efficacy [20]. To determine the impacts of the education on clinical endpoints, HbA1c values were measured postpartum. HbA1c values were approximately 23% and 27% lower in the intervention group three and six months after the intervention, respectively [20]. Concerning the health belief constructs, the intervention significantly improved perceived susceptibility, severity, benefits, self-efficacy, cues to action, and self-management between baseline and six months after the intervention by 40%, 34%, 28%, 40%, 84%, and 40%, respectively [20]. The study also reported a statistically significant reduction from baseline in perceived barriers six months after the intervention by 147% [20]. These data along with the HbA1c reductions indicate an increased capacity in women with GDM to maintain healthy lifestyle modifications and self-management skills postpartum when provided DSME rooted in the health belief model and additional support through phone call check-ins.

The three studies that assessed 2 h postprandial glucose (2hPPG) and fasting plasma glucose (FPG) each included a component of online or digital support to complement the structured health education [15,17,21]. The first study that assessed 2hPPG and FPG integrated personalized psychological nursing with structured health education to navigate anxiety, depression, and other negative emotions [15]. The health education was provided to both the control and intervention groups, but the intervention group also received *WeChat* online communication to continue the education beyond the facility to answer questions and provide physical activity recommendations. The intervention resulted in a statistically significant lower 2hPPG and FPG than the control group, which was a 12% and 14% decrease from the baseline [15]. The second study also organized a *WeChat* group that included the healthcare team, patients, and patients’ family members to continue education and self-management, facilitate shared dialogue, and allow for questions to be answered in a timely manner [17]. This intervention continued until the end of the day before delivery. Both the intervention and control groups reported a reduced 2hPPG and FPG from the baseline (49% and 47% reduction for the intervention group and 40% and 26% reduction for the control group, respectively) [17]. Although the control group also reported a reduction in blood glucose values, the intervention values were lower than the control group by approximately 11% (2hPPG) and 25% (FPG), which were statistically significant [17]. The study also reported outcomes related to self-management, and the intervention arm experienced a statistically significant improvement in ability and attitude [17]. The final study provided a self-care guidebook, logbook, and educational software to complement the group-based education lessons [21]. This software included lifestyle components (e.g., nutrition and physical activity) as well as relaxation and stress-management techniques [21]. The intervention was provided to women up to 34 weeks gestation [21]. The study reported a statistically significant 19% reduction in 2hPPG between the intervention and control group, and the intervention elicited an approximate 34% reduction from the baseline [21]. Interestingly, the intervention did not yield a statistically significant improvement in FPG compared to the control group [21]. Self-efficacy improved in the intervention group by approximately 164%, which was statistically significant and greater than the control group by 69% [21].

Similarly, the two studies measuring glycemic control both used complementary technology interfaces to increase engagement with the subjects and continue the education and self-management practices [16,22]. The first study implemented a *WeChat* group communication platform to provide weekly self-management encouragement in addition to routine DSME care [16]. The *WeChat* communication allowed patients to engage with the healthcare team and learn through peer interactions by sharing recipe ideas and experiences [16]. The intervention was provided until delivery, and then, blood glucose values were recorded at certain times throughout the day (five readings per day) for six days within two weeks for a set number of intervals determined by enrollment and delivery gestational age [16]. The primary outcome, glycemic qualification rate, was calculated as the number of blood glucose values within the control range divided by 30 (the total number of readings in a two-week interval) and multiplied by 100% [16]. Blood glucose was collected while fasting, before sleeping, and two hours after breakfast, lunch, and dinner [16]. Results were reported based on the gestational age of enrollment and those who began the intervention earliest (i.e., 23–24 weeks) reported a nearly 22% improvement in glycemic qualification rate compared to the control group at the first blood glucose measurement interval [16]. After one month of the intervention, the glycemic qualification rate was greater than the control group for those enrolled between 23 and 26 gestational weeks by an average of 23% [16]. The subjects enrolled between 27 and 30 gestational weeks did not report statistically different glycemic qualification rates compared to the control groups [16]. These findings suggest that early intervention with DSME can improve blood glucose management in women with GDM. The second study incorporated a web-based interface to provide additional information related to the topics covered in the standard education class [22]. In particular, this web platform included extra information on healthy foods and grocery shopping tips and offered a quiz at the end of each session to assess understanding [22]. The intervention yielded a statistically significant reduction in glycemic levels from baseline (45%), although both the control and intervention groups returned to normal glycemic levels six weeks postpartum [22]. The intervention did reduce the subjects’ body mass index (BMI); however, at 12 weeks postpartum, approximately half of the subjects were above their pre-intervention weight and categorically overweight or obese [22].

The remaining studies assessed the 2 h oral glucose tolerance test (OGTT) postpartum after intervening with health education programs that were either supplemented with a smartphone application [19] or weekly phone calls [23]. Both studies continued the education postpartum and assessed 2 h OGTT postpartum to evaluate the relationship between management education and the body’s ability to process large glucose loads. The first study provided additional nutrition information including culturally adapted recipes for food and drink via a smartphone application [19]. Despite the education continuing postpartum through the application, the control and intervention groups did not report statistically different 2 h OGTT values during the postpartum measurement [19]. Similarly, the intervention did not yield a statistically different postpartum 2 h OGTT result from baseline when compared to the control group [19]. The secondary outcomes of this study included medication use for GDM and delivery-related measurements (e.g., labor induction, Apgar score, and transfer to a neonatal intensive care unit) [19]. Statistically significant differences between the control and intervention groups were not reported for these secondary measurements [19]. The second study adapted a diabetes prevention program to elicit behavior change for GDM self-management and incorporated two nutrition counseling sessions, physical activity encouragement, and weekly phone calls to review the chapters of the education manual [23]. Data were collected 6.4 ± 2 weeks postpartum for the intervention group, and the 2 h OGTT values were not statistically different from the control group [23]. Insulin resistance, as measured by homeostatic model assessment for insulin resistance (HOMA-IR); FPG; and 2hPPG were also not statistically different between the intervention and control groups [23]. The intervention group was stratified by weekly phone call compliance, but no statistical difference was found between compliance and glucose control as measured by fasting or 2hPPG levels [23]. Total gestational weight gain and weight gain by trimester were reported; however, the intervention did not have a significant impact on these outcomes [23]. Based on these reported findings, DSME interventions, even with supplemental supports, do not appear to impact long-term blood glucose metabolism as measured by the 2 h OGTT.

### 3.2. Medical Nutrition Therapy

As Table 2 indicates, three clinical trials assessed clinical endpoints related to MNT interventions [24,25,26]. All the interventions provided personalized nutrition therapy to align with MNT as defined by the Nutrition Care Process [10]. Various topics, such as food exchange, blood glycemic load, and macronutrient distribution, were incorporated in the education of the patients to continue the nutrition-related behaviors. In addition to nutrition, some interventions incorporated exercise primarily through recommendations for postprandial engagement [24,25].

Of the two studies that assessed 2hPPG, the first study kept subjects on site for 12 h to provide MNT counseling, diabetes education, and supply three meals and two snacks constructed based on the tailored nutrition needs of the subject [24]. After the 12 h intervention, the subjects had access to a *WeChat* platform to continue communication with the healthcare team and their peers [24]. The intervention reduced 2hPPG by approximately 27% in subjects receiving MNT counseling and intensive education [24]. When compared to the control group, 2hPPG was reduced approximately 10% more after lunch and 6% more after dinner in the intervention group, which were both statistically significant [24]. The study also reported a statistically significant lower average gestational weight gain in the intervention group compared to the control group (0.3 kg compared to 0.5 kg) [24]. The second study provided personalized nutrition therapy based on the concept of blood glycemic load and compared this intervention to personalized nutrition therapy based on traditional food exchange methods [25]. After two weeks of the intervention, approximate 26% and 24% decreases in 2hPPG and FPG, respectively, were reported, both of which were statistically significant [25]. Although not statistically significant, the food exchange method still reduced 2hPPG and FPG by 20% and 17%, respectively [25].

The final study incorporated varying macronutrient ranges in the MNT counseling based on pre-pregnancy weight [26]. The intervention group received 20% protein, 40% carbohydrates, and 40% fat dispersed between three meals and three snacks [26]. The intervention resulted in a significant 0.9 kg less gestational weight gain when compared to the control group; however, this difference dematerialized when the results were adjusted for follow-up timing [26]. These findings suggest that this particular macronutrient distribution does not have a statistically significant impact on gestational weight gain in women with GDM [26].

Of the studies that assessed birth-related complications, 12 h intensive MNT and education counseling yielded a statistically significant reduced incidence of gestational hypertension and preterm labor [24]; however, the MNT intervention based on blood glycemic load did not yield a similar reduction [25]. The blood-glycemic-load-based MNT also did not impact fetal macrosomia, eclampsia, or fetal distress [25].

## 4. Discussion and Conclusions

### 4.1. Discussion

While MNT and personalized nutrition therapy are considered fundamental to the standard of care for diabetes management [27], we found similar reductions in FPG and 2hPPG between MNT and DSME interventions for GDM. The results of this review demonstrate that DSME and MNT reduce 2hPPG throughout the third trimester and across baseline 2hPPG values ranging from 7.73 to 11.70 mmol/L (~140 to 210 mg/dL). Our review similarly demonstrates benefits from DSME and MNT in reducing FPG. All the studies that intervened in subjects with a baseline FPG above the normal range (>5.6 mmol/L, >100 mg/dL) reported improvements after the interventions. Of the clinical endpoints assessed, gestational weight gain was the least impacted by both DSME and MNT interventions, suggesting that blood glucose management in GDM is achievable independent of gestational weight management. These findings align with a recent study reporting that changes in gestational body weight only contributed to 9% of changes in insulin sensitivity during pregnancy [28]. When comparing the two intervention types, it is imperative to note that all the DSME interventions included some form of continued complementary support beyond structured health education. This support included web-based, smartphone, and messaging applications to increase engagement. The control groups in the MNT studies often received traditional structured health education that included nutrition topics. Additional research is needed to better establish the differences between technology-supported DSME interventions and MNT in women with GDM.

Even though only a few MNT clinical trials were identified through this scoping review, retrospective analyses on women with GDM have also indicated benefits to blood glucose control. In a retrospective analysis comparing MNT to generic health education, Zhang et al. reported a 38% reduction from the baseline in FPG [29]. The personalized nutrition also yielded lower HbA1c levels than the control group at eight months, nine months, during labor, and one month postpartum [29]. In a second retrospective cohort study, MNT counseling resulted in 2hPPG and FPG values 37% and 18% lower than the control group’s values [30]. Shi et al. also assessed gestational weight gain and reported a fluctuation in weight gain from GDM diagnosis to delivery [30]. These findings support the findings of our review and suggest that MNT interventions have a substantial impact on glucose metabolism, regardless of the effect on gestational weight gain. Prospective cohort studies have similarly reported that excessive weight gain does not significantly differ between women with GDM receiving MNT and borderline glucose-intolerant women not receiving MNT [31].

In addition to nutrition, the self-monitoring of blood glucose is a valuable component of structured health education for diabetes management [32]. All the DSME interventions included blood glucose self-monitoring education, compared to only two of the MNT studies. One of the two MNT interventions with a self-monitoring component did not report blood glucose outcomes, thus eliminating the ability to compare MNT with self-monitoring education to MNT alone. Future studies should compare MNT interventions with additional self-monitoring education to assess for improved outcomes.

With respect to the underlying frameworks used in the interventions, self-efficacy was the most common behavior change construct among the included studies. Self-efficacy is associated with nutrition habits, exercise, and self-monitoring blood glucose practices in patients with T2DM [33]. A multivariate model showed that with each 10% increase in self-efficacy scores, T2DM patients were more likely to report optimal diet, exercise, and self-monitoring blood glucose practices [33]. Similarly, Al-Hashmi et al. reported that self-efficacy education for women with GDM improved adherence to diet, physical activity, and blood glucose testing recommendations [34]. The DSME interventions included in our review improved self-efficacy scores as well as blood glucose values. Future intervention designs should continue to include self-efficacy as a core component to improve the capacity of women with GDM to change behavior and adhere to recommended diabetes care.

The design of nutrition-related GDM interventions must also incorporate the cultural and educational context of its recipients. As lifestyles vary globally, DSME and MNT interventions must consider these differences. Food selection and eating patterns are strongly intertwined with culture and nutrition recommendations must adapt to these relationships. Food exchanges and carbohydrate counting are common ways to educate about food selection in DSME and in MNT interventions. Food exchange lists and resources must be tailored to provide culturally relevant options [35]. The cultural perspective is of utmost importance to the effectiveness of food exchange lists [35]. For eating patterns, small, frequent (five to six) meals were recommended throughout the articles included in our review. Some cultures, however, have a large meal in the afternoon with two smaller meals in the morning and evening [36]. The recommended small meal eating pattern may oppose the cultural or lifestyle norm of a community. Similarly, religious observations of fasting may challenge the ability to evenly time carbohydrate intake throughout the day [36]. These considerations are critical to the success of an intervention and should be explored further in future reviews.

Key findings from our review highlight the efficacy of technology-supported DSME and MNT interventions in promoting normoglycemia. GDM diagnosis and management are important predictors of maternal and fetal health during childbirth and later in life. Fetal exposure to hyperglycemia has potential epigenetic implications for future chronic disease or metabolic dysfunction [37]. In particular, intrauterine exposure to GDM contributes to 47% of T2DM in offspring, implying a strong association between the two [38]. In animal models, high-glucose environments have altered mouse placental gene expression resulting in the hypermethylation, up-regulation, and down-regulation of imprinted genes [39]. These alterations in expression can disrupt the proteins that promote the activation of the insulin/insulin-like growth factor, thus increasing the potential for diabetes onset in offspring [39]. These reported improvements in hyperglycemia from DSME and MNT interventions may suggest a reduced risk of diabetes mellitus development for offspring and maternal progression to T2DM.

### 4.2. Strengths and Limitations

The present review has multiple strengths. First, the authors examined the dearth of GDM and nutrition research by reviewing the current literature to compare two intervention methods (i.e., DSME and MNT) which are the recommended guidelines of care for diabetes management. The literature review followed a structured search and focused selection processes based on eligibility criteria. Through the search, the authors were able to identify the current gap in clinical trials related to nutrition-education-based interventions for GDM management. Secondly, the authors assessed the application of DSME, a service more commonly integrated into T2DM care, in GDM management. Thirdly, many of the studies assessed the same clinical endpoints, which allowed for comparison between the two intervention categories.

The limitations of the review include the omission of a statistical analysis on the average result of the data from the separate studies due to the variability in study designs. Despite this, the review aimed to provide readers with an overview of outcomes related to DSME and MNT in cases of GDM. Second, only four platforms were utilized for the literature search; however, all are considered major repositories of peer-reviewed and published health and nutrition clinical trials. The selected articles had to be available in English, which may have also limited the scope of this review. The methodological limitations of the studies that were part of our review included varying levels of detail related to the DSME interventions, especially about nutrition. This variation in detail diminished the ability to assess the significance of nutrition in the reported outcomes when compared to other components in the intervention. Additionally, the subjects were not assessed for long-term behavior change outcomes, hindering the generalizability of these interventions and reducing the future risk of GDM or T2DM progression. Finally, while two interventions designed culturally adapted nutrition education, the findings from this review are limited by cultural constraints. The interventions that improved GDM blood glucose management are best suited for populations such as those included in the study. Future studies should continue to expand on culturally relevant DSME and MNT interventions to account for the global differences in educational processes and lifestyle.

### 4.3. Future Directions

Our findings suggest that DSME interventions with complementary technology-based supports have comparable outcomes to MNT interventions for patients with GDM. While personalized and individualized nutrition therapy may be the gold standard of diabetes management, this review indicates that benefits still exist with the general nutrition education and heterogeneity found in DSME. Although these findings are promising, only a limited number of studies were available for GDM compared to nutrition-based interventions for T1DM and T2DM. We recommend that future nutrition-based interventions are developed intentionally for GDM, rather than adapted from T2DM care, and evaluated for both long-term and short-term outcomes. In addition, these interventions should be mindful of the cultural context of its recipients.

### 4.4. Conclusions

**Figure 2:**
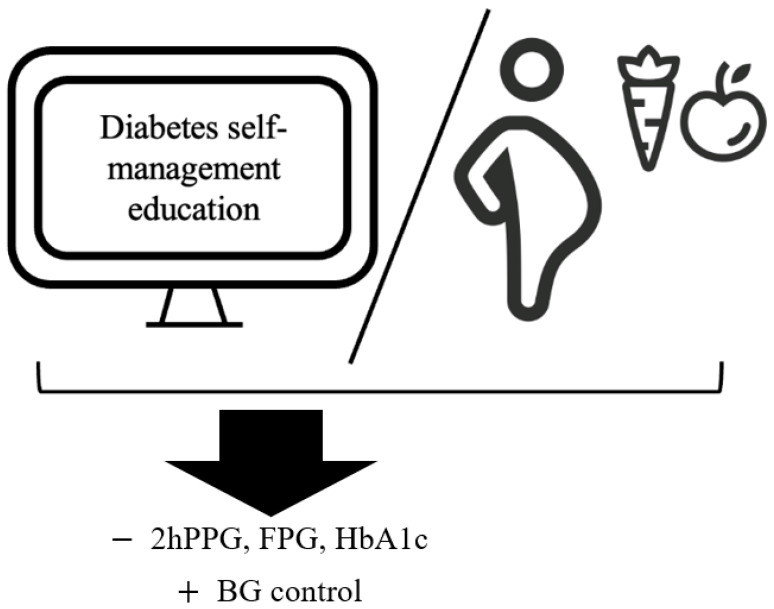
Effects of technology-supported diabetes self-management education and medical nutrition therapy on gestational diabetes clinical endpoints. − indicates reduction; + indicates improvement; 2hPPG: 2 h post prandial plasma glucose; BG: blood glucose; FPG: fasting plasma glucose; HbA1c: glycated hemoglobin.

Our scoping review identified 12 studies that assessed the impacts of integrating nutrition in DSME and MNT on clinical endpoints in subjects with GDM. This analysis aims to inform readers on the current literature and gaps in the literature related to nutrition-education-based interventions for GDM. In addition, the review compares generic nutrition education to individualized and personalized nutrition therapy (MNT). Although the nutrition education in each DSME intervention varied, the outcomes suggested similar management of hyperglycemia to MNT (Figure 2). That said, the heterogeneity of the DSME interventions makes it challenging to assess the impacts of nutrition compared to other integrated factors (e.g., the self-monitoring of blood glucose). With the increasing prevalence of GDM worldwide, the development of DSME interventions with nutrition education that yield similar outcomes to MNT interventions for GDM management should be further explored. Successful DSME interventions may increase the accessibility of care since many healthcare professionals can deliver the information, and continued support can be integrated into daily life. Hyperglycemic management in GDM may have profound impacts on maternal health and the health of future generations, thus warranting future effective nutrition-related investigations.

## Figures and Tables

**Table 1:** Studies measuring the outcomes of diabetes self-management education in women with gestational diabetes mellitus.

Author, Year (Country)	Study Design	Gestational Age at Intervention	DSME Intervention	Nutrition Education Content of DSME	Assessment of Clinical Endpoints
He et al., 2022 (China) [15]	RCT (*n* = 170)	28.3 ± 4 wks	Structured HE; exercise; BG self-monitoring; personalized psychological nursing; PA	Low-fat, high-fiber, low-glycemic diet	↓ 2hPPG, FPG
Tian et al., 2021 (China) [16]	RCT (*n* = 269)	23–30 wks	Weekly management/HE messages through *WeChat*; BG self-monitoring; PA	Increasing food diversity, high-fiber cereals; recipes; photos of meals; PA	↑ BG control
Guo et al., 2021 (China) [17]	RCT (*n* = 140)	26.7 ± 3 wks	Integrated doctor and nurse communication; *WeChat* HE communication for families and patients; PA	Five to six meals/day; avoid high sugar, oil and fat; avoid a spicy diet; prioritize F/V	↓ 2hPPG, FPG ↑ self-management ability
Yew et al., 2021 (Singapore) [18]	RCT (*n* = 333)	27 ± 3.2 wks	Structured HE based on HBM; smartphone app with more detailed dietary/PA guidance	CHO and kcal amounts of culturally relevant foods	↓ proportion of BG above targets
Borgen et al., 2019 (Norway) [19]	RCT (*n* = 233)	<33 wks	Structured HE; exercise; BG self-monitoring; smartphone app with more detailed diet/PA recommendations, feedback on BG	Culturally adapted diet suggestions; limited sugar; increased WG, F/V; small meals	∅
Mohebbi et al., 2019 (Iran) [20]	RCT, quazi-experimental (*n* = 110)	Not provided	Structured HE based on HBM; BG self-monitoring; phone call reminders; motivational interviewing	Healthy diet	↓ HbA1c
Kolivand et al., 2019 (Iran) [21]	RCT (*n* = 151)	26.7 ± 5 wks	Structured HE up to 34 wks GA; self-care guidebook, log book; educational DVDs; stress reduction; PA; BG self-monitoring	Nutrition assessment and control	↓ 2hPPG
Carolan-Olah et al., 2019 (Australia) [22]	RCT (*n* = 110)	28–32 wks	Structured HE; web-access to HE with quizzes; healthy habits, lifestyle; emotions; BG self-monitoring	Healthy food choices; healthy shopping; increase V; identify sugar	↓ BMI, SBP, glycemic level
Durnwald et al., 2016 (United States) [23]	RCT (*n* = 101)	30.8 ± 2 wks	Structured behavioral modification HE; motivational messaging; weekly phone call review; pedometer for PA; BG self-monitoring	ADA dietary guidelines; nutritional facts	∅

Down arrow (↓) indicates reduction. Up arrow (↑) indicates increase. Crossed circle (∅) indicates no statistically significant impact of clinical endpoints. 2hPPG: 2 h post prandial plasma glucose; ADA: American Diabetes Association; BG: blood glucose; BMI: body mass index; CHO: carbohydrate; F/V: fruits and vegetables; FPG: fasting plasma glucose; HbA1c: glycated hemoglobin; HBM: health belief model; HE: health education; kcal: kilocalories; PA: physical activity; RCT: randomized controlled trial; SBP: systolic blood pressure; wks: weeks.

**Table 2:** Studies measuring the outcomes of medical nutrition therapy in women with gestational diabetes mellitus.

Author, Year (Country)	Study Design	Gestational Age at Intervention	MNT Intervention	Assessment of Clinical Endpoints
Yuan et al., 2020 (China) [24]	RCT (*n* = 312)	24–28 wks	12 h comprehensive nutrition care process; five meals individualized to personal needs over 12 h; education on food exchange method and calorie counting	↓ 2hPPG, FPG, GWG, incidence of complications
Lv et al., 2019 (China) [25]	RCT (*n* = 134)	35 wks	Personalized diet based on ideal weight, actual weight gain, eating habits; education based on blood glycemic load	↓ 2hPPG, FPG; ∅ incidence of complications
Moreno-Castilla et al., 2013 (Spain) [26]	RCT (*n* = 130)	30.4 ± 3 wks	Caloric intake calculated based on pre-pregnancy body weight; 20% PRO, 40% CHO, 40% fat; design of three meals and three snacks per day divided by CHO distribution	∅

Down arrow (↓) indicates reduction. Crossed circle (∅) indicates no statistically significant impact of clinical endpoints. 2hPPG: 2 h post prandial plasma glucose; CHO: carbohydrate; FPG: fasting plasma glucose; GWG: gestational weight gain; PRO: protein; RCT: randomized controlled trial; wks: week.
